# From signal to construct evidence: multimodal consumer-wearable sensing in real general-aviation flight

**DOI:** 10.3389/fnrgo.2026.1911259

**Published:** 2026-09-01

**Authors:** Rongbing Xu, Shi Cao, Michael Barnett-Cowan, Gulnaz Bulbul, Suzanne Kearns, Ewa Niechwiej-Szwedo

**Affiliations:** 1 Department of Systems Design Engineering, University of Waterloo, Waterloo, ON, Canada; 2 Waterloo Institute for Sustainable Aeronautics, University of Waterloo, Waterloo, ON, Canada; 3 Department of Kinesiology and Health Sciences, University of Waterloo, Waterloo, ON, Canada; 4 Department of Geography and Environmental Management, University of Waterloo, Waterloo, ON, Canada

**Keywords:** construct validity, ecological validity, eye movements, general aviation, heart rate, neuroergonomics, psychological stress, wearable sensors

## Abstract

We asked how far multimodal consumer-wearable signals recorded during real general-aviation flight can be treated as construct-relevant measurement rather than as records of motion, posture, or task structure. Forty pilots flew five standardized maneuvers in a Cessna 172 while frontal electroencephalography (EEG and AF7/AF8), cardiac activity, electrodermal activity (EDA), eye movements, skin temperature, and flight dynamics were recorded, with segments paired with pilot self-reports and the four in-air maneuvers additionally paired with instructor competency ratings. We assembled three nested tiers of evidence, namely, known-groups sensitivity, within-person convergent evidence, and criterion-related association, and then tested prediction in held-out pilots. Signal quality supported inference, with trial-level availability from 85% to 97%. Self-reported stress varied across maneuvers (*H* = 61.1), but because maneuver order was largely fixed this variation is confounded with elapsed time, fatigue, physical dynamics, and sensor settling, and therefore cannot establish that any signal is specific to stress. Within-person convergence was strongest for cardiac (mean heart rate *r*_rm_ =.57) and oculomotor (saccade and fixation rates *r*_rm_ =.39) channels, with phasic EDA and frontal theta adding weaker support. Criterion-tier associations were re-examined with leave-one-pilot-out and wild cluster bootstrap inference: Kinematic and electrodermal associations held, whereas every frontal theta/alpha association proved to rest on individual pilots and did not survive. In cross-validation with tuning nested inside each training fold, sensor features did not exceed a maneuver-only baseline across pilots. Low-cost cardiac and oculomotor channels are the most promising basis for within-person stress monitoring, while pilot-general prediction will require individual calibration, stronger reference measures, and larger samples.

## 1 Introduction

Neuroergonomics studies the brain at work, bringing the methods of neuroscience and physiology to bear on human performance in realistic tasks and environments (Parasuraman, 2003; Mehta and Parasuraman, 2013). A recurring tension is that its constructs (cognitive states such as mental workload, stress, and situation awareness) matter most in operational settings yet are most often measured where conditions can be controlled (Charles and Nixon, 2019; Wickens, 2008; Endsley, 1995b). Aviation illustrates the problem. Workload and situation awareness are central to flight safety and to competency-based training, and a long tradition of psychophysiology has linked them to electroencephalographic (EEG) features, cardiac activity, electrodermal activity (EDA), and eye-movement patterns (Roscoe, 1992; Hankins and Wilson, 1998; Wilson, 2002; Hughes et al., 2019; Peißl et al., 2018; Luzzani et al., 2024). Carrying that evidence into the cockpit itself, rather than a simulator, has been slow because research-grade instrumentation is difficult to deploy during real flight (Veltman, 2002; Johannes et al., 2017; Fuentes-García et al., 2021). A small number of studies have nonetheless recorded neural signals in flight. For example, Dehais et al. (2019) monitored pilot workload with a dry-electrode EEG system during real flight and found spectral-power differences between load conditions, even though single-trial classification did not exceed chance.

Consumer-grade wearable sensors widen what is logistically possible. Self-applied, wireless, and inexpensive devices can record EEG, cardiac, electrodermal, eye, and flight-dynamics signals simultaneously in an operating aircraft (Peake et al., 2018; Carrier et al., 2020; Dudarev et al., 2023; Hu et al., 2024). Individual devices have been validated against laboratory references under controlled conditions (Gilgen-Ammann et al., 2019; Schaffarczyk et al., 2022; Krigolson et al., 2017; Cannard et al., 2021; Milstein and Gordon, 2020). Feasibility is not the same as validity. A device can produce a clean, physiologically plausible recording that still fails to index the cognitive construct a study cares about, because the signal is dominated by motion, posture, or task structure rather than by cognitive state. For field neuroergonomics, the substantive question is therefore not only whether these signals can be captured, but how far they behave as construct-relevant measurement of cognitive state and its behavioral and performance correlates.

We frame this question through construct validity in the classical sense (Cronbach and Meehl, 1955). Adequate signal quality is a necessary precondition: A signal that is not captured cannot index anything. It is not sufficient. Gathering construct-relevant evidence for a field measurement requires showing that it behaves as the construct should: that it varies with conditions expected to move the construct, that it covaries within individuals with other indicators of the same state, and that it relates to outcomes the construct should predict. These map onto three nested questions for cockpit sensing. First, do the maneuvers and the rating instruments produce structured variation in the outcome labels, rather than noise (known-groups sensitivity)? Second, do sensor features track self-reported state within pilots, where between-person differences are removed (convergent evidence)? Third, do features explain instructor-rated performance after adjusting for the pilot and the maneuver (criterion-related association)? Each tier is weaker in the field than in the laboratory, and we treat none of them as sufficient on its own. A field protocol cannot manipulate one construct while holding the others fixed, so known-groups variation is graded by whatever covaries with the maneuver sequence, not by demand alone; the reference measures available in a training cockpit are themselves imperfect indicators rather than criterion standards; and the sample sizes that real flight permits leave the criterion tier fragile. Our aim is accordingly to establish how far the evidence currently reaches and where it stops, not to certify any modality as a validated index of stress or workload. The nested logic itself is not new, and concurrent-criterion protocols for wearable signals are established (van Lier et al., 2020). What is uncommon is applying it inside a real cockpit and then testing the predictive boundary directly.

One EEG feature warrants attention in advance because it carries part of the neural argument. The frontal theta/alpha ratio combines two effects that move in opposite directions with cognitive demand. Frontal theta tends to increase with engagement, cognitive control, and working-memory load (Cavanagh and Frank, 2014; Klimesch, 1999), while alpha tends to desynchronize with attentional investment (Klimesch, 2012). The ratio should therefore rise when demand increases, and band ratios of this kind have been related to self-reported workload (Raufi and Longo, 2022). We measured frontal theta and alpha from lateral frontal sites (AF7, AF8), not from the midline (Fz) where frontal midline theta is classically recorded, so we treat the ratio as a lateral-frontal spectral summary rather than as a direct index of midline theta sources. We state the expectation here because the data did not bear it out: the ratio proved too unstable in this recording setting to support any conclusion, which is itself worth reporting for a field measure that the literature would lead one to expect much of.

**Figure 1:**
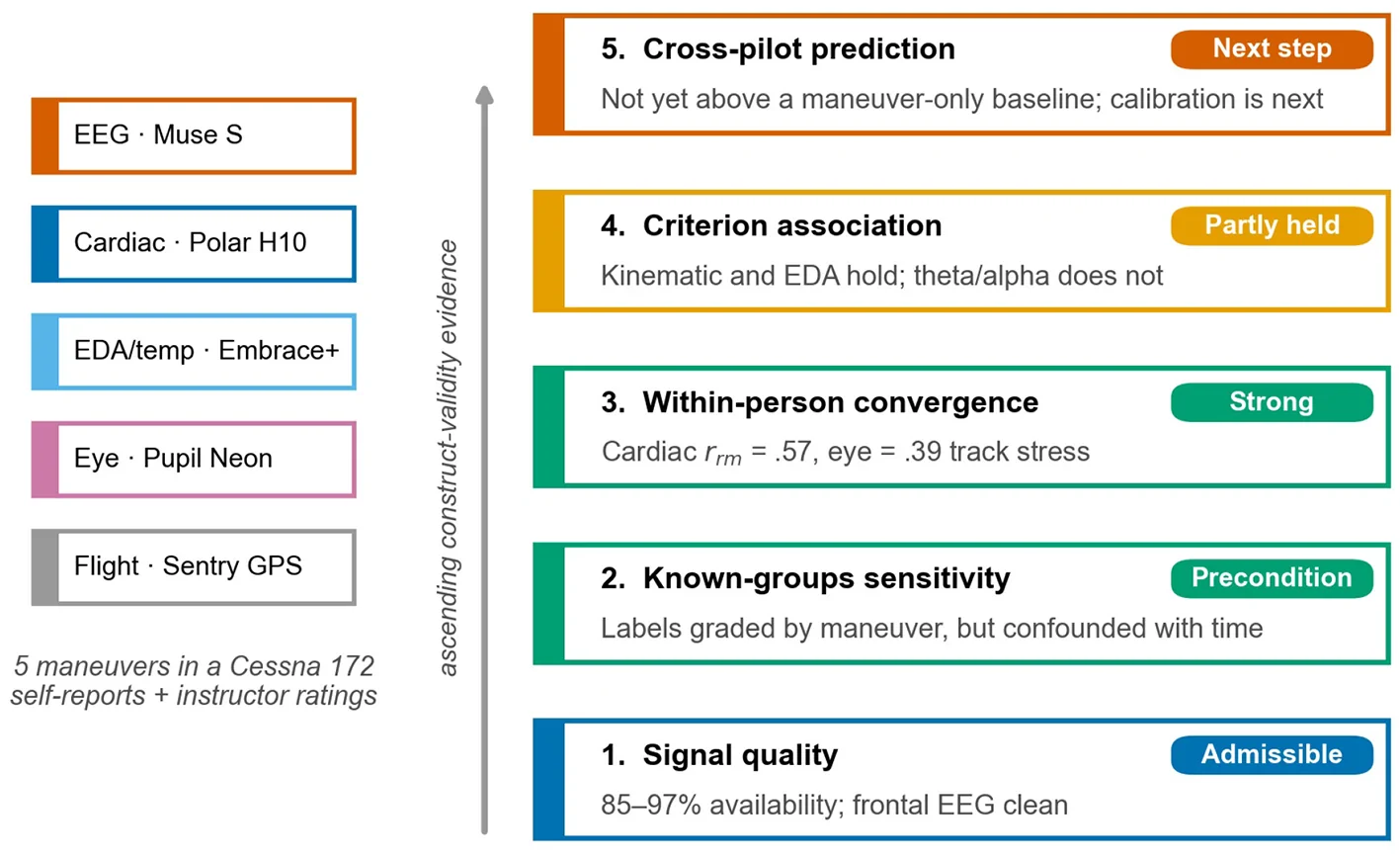
Study design and the evidence ladder. Five consumer-grade sensor systems recorded simultaneously during five standardized maneuvers in a real Cessna 172, paired with pilot self-reports and instructor competency ratings. Evidence was built as an ascending ladder, from signal quality through known-groups sensitivity, within-person convergent evidence, and exploratory criterion-related association, to an explicit cross-pilot predictive-boundary test, with a one-line takeaway at each rung.

The present study uses multimodal recordings from 40 pilots flying five standardized maneuvers in a real general-aviation aircraft. To our knowledge, it is the first study to record synchronized, consumer-grade multimodal physiology during real general-aviation flight for research, so the in-flight dataset and its quality characterization are themselves a contribution. We treat signal quality as an enabling foundation and report it in full, then build the three tiers of construct-relevant evidence on top of it. We also test that evidence directly with cross-pilot cross-validation, asking whether the sensor features already support generalizable prediction in held-out pilots, a deliberately stricter target than within-sample association. The broader contribution is a measurement-grounded account of how far consumer-grade neuroergonomic sensing can be taken in the wild today, and of the concrete directions the evidence marks out as most promising, weighed across modalities rather than around a single marker. Figure 1 summarizes the design and the nested evidence structure.

## 2 Materials and methods

### 2.1 Participants and design

Forty pilots were recruited from two flight training organizations in Ontario, Canada, namely, the Brantford Flight Centre and the Waterloo Wellington Flight Centre. The sample comprised 35 men and 5 women, with a median age of 23 years (range 18–78) and a median of 155 logged flight hours (range 20–1,800). They held private (*n* = 23), commercial (*n* = 11), or student (*n* = 6) pilot certificates. Each flew five standardized segments in a Cessna 172 under visual flight rules, accompanied by a certified flight instructor, in a largely standard order: a seated ground Baseline recorded before engine start, Normal Takeoff, Steep Turn (45-degree bank), Power-on Stall (entry and recovery), and Normal Landing. Instructors occasionally adjusted the sequence of the in-air maneuvers in response to weather and traffic conditions. The full protocol, including recruitment, device integration, preprocessing, and the competency-based instructor assessment, is documented by Xu et al. (2025). Companion analyses of the same cohort have separately examined peripheral cardiac, electrodermal, and thermal signals (Xu et al., 2026a), participant-independent frontal-EEG classification (Xu et al., 2026b), and cross-modal physiological coupling (Zahorska et al., 2026). The present study draws on the same recordings but differs from each of them in the modalities entered, the outcomes modeled, the trial segmentation, and the statistical approach; Table 1 sets out the comparison in full, and no analysis, table, or figure reported here appears in any of the companion papers. A sixth composite segment (a full traffic-pattern Circuit) was part of the original protocol but is excluded here: It was the longest and most heterogeneous segment and was not flown by all pilots, and its duration confounds maneuver-specific effects with elapsed recording time. Restricting analysis to the five standardized maneuvers gives a 40 × 5 = 200 nominal trial-participant design. Because the maneuvers generally followed this order, maneuver identity is largely confounded with elapsed flight time, fatigue, and sensor settling. The occasional weather-driven reordering provides only limited deconfounding, a point we return to in the Limitations.

**Table 1:** Relationship of the present study to the companion publications drawing on the same pilot cohort.

Study	Modalities analyzed	Outcomes and Segmentation	Statistical approach
Xu et al. (2026a) (Sensors)	Peripheral only: cardiac, EDA, skin temperature. No EEG, eye, or flight dynamics	Binary state labels; windowed sub-trial epochs	Leave-one-subject-out machine-learning classification
Xu et al. (2026b) (IEEE Sens. J.)	Frontal EEG only	Binary state labels; windowed EEG epochs	Participant-independent classification; different author set
Zahorska et al. (2026) (ICASSP)	Pairwise cross-modal signal coupling	Continuous signal streams, no rating outcomes	Granger-causality coupling analysis
**Present study**	All six streams jointly: EEG, cardiac, EDA, eye, temperature, flight dynamics	Whole-maneuver trial-level features; self-reports and instructor competency ratings as outcomes; five standardized maneuvers, Circuit excluded	Known-groups tests, within-person repeated-measures correlation, fixed-effect criterion models with bootstrap small-cluster inference, feature-selection sensitivity analyses, grouped cross-validation

All four share the recruitment, instrumentation, and raw acquisition of the protocol paper (Xu et al., 2025), and diverge thereafter. The three tiers of evidence, the small-cluster robustness analyses, the cross-pilot vs. within-subject comparison, and the signal-quality characterization are unique to the present manuscript, and no table or figure is shared with any companion paper.

### 2.2 Constructs and operationalization

Three cognitive constructs were operationalized through converging indicators. Mental workload, acute stress, and situation awareness were captured by brief 0–10 pilot self-reports. Acute stress was rated for all five segments, whereas workload and situation awareness were rated only after the four in-air maneuvers, so the stress ratings cover 200 trials and the workload and situation-awareness ratings cover 160. These were compact task-level ratings rather than substitutes for full NASA-TLX or query-based situation-awareness measures (Hart and Staveland, 1988; Endsley, 1995a; Taylor, 2017), and single-item measures carry limited reliability, which attenuates correlations. Instructors independently rated performance on a standardized competency-based rubric (Xu et al., 2025), yielding technical and non-technical performance scores together with item-level ratings of instructor-observed situation awareness, workload management, problem-solving, and teamwork. The rubric followed the competency-based training and assessment approach standard in Canadian ab-initio instruction (Luzzani et al., 2024), scoring each competency on ordinal anchors. Each flight was rated by its single accompanying instructor, so inter-rater reliability could not be estimated here, and the ordinal ratings show ceiling effects; both attenuate the criterion associations. Competency-based ratings of pilot non-technical skills carry only moderate reliability even among trained examiners [test–retest near 0.6, inter-rater near 0.5; (Gontar and Hoermann, 2015)], which further bounds the criterion tier. Physiological and behavioral indicators were derived from the sensor recordings (Section 2.3). Treating self-reports, instructor ratings, and sensor features as three partly independent operationalizations makes the tiered logic tractable.

These three sources sit at different distances from the constructs and are not interchangeable. The self-reports measure *reported state*: what a pilot was willing and able to say about their own experience immediately after a maneuver, on one item per construct. The instructor ratings measure *observed performance*: what a single accompanying instructor judged from the right seat, on ordinal competency anchors, while also flying with the participant. Neither measures the latent construct. Workload, stress, and situation awareness are inferred states that no instrument available in a training cockpit observes directly, and both reference measures are imperfect indicators of them, the self-reports through single-item unreliability and the instructor ratings through ceiling effects, ordinal coarseness, and the absence of a second rater. We therefore read agreement between a sensor feature and one of these measures as evidence that the feature tracks that indicator, not as validation against a criterion standard, and we report disagreement between the two reference sources, as for situation awareness, rather than privileging either. The strongest claim this design supports is convergence among fallible indicators.

### 2.3 Multimodal sensing

**Figure 2:**
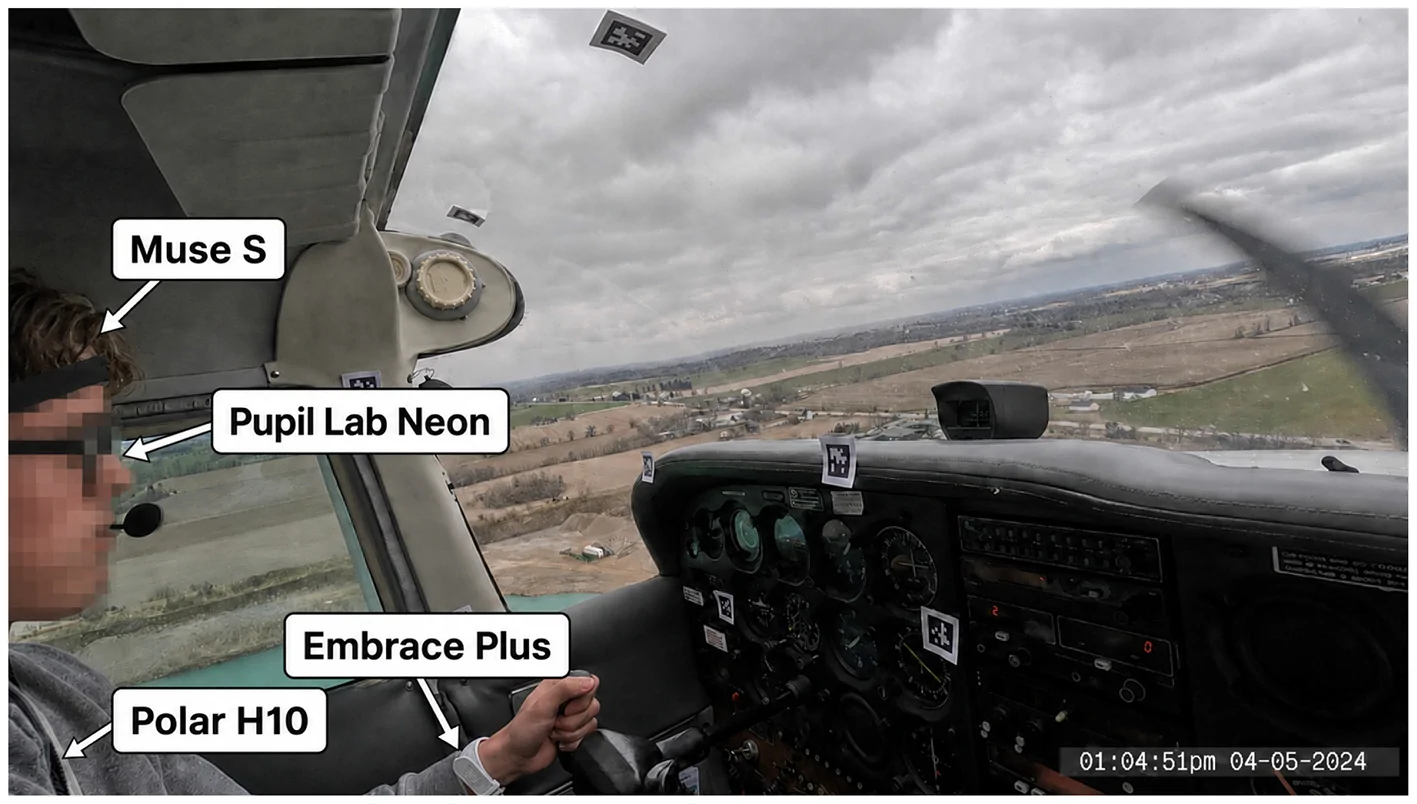
Wearable sensor configuration in the Cessna 172 cockpit, on a participating pilot during flight (face obscured for privacy). Four of the five devices are visible and labeled: the Muse S headband (frontal EEG), the Pupil Labs Neon glasses (eye movements), the Embrace Plus wristband (EDA and skin temperature), and the Polar H10 chest strap (cardiac activity). The Sentry Plus GPS recorder was attached to the side window behind the participant and is out of view. The AprilTag markers supported gaze mapping.

Five consumer-grade devices recorded simultaneously (Table 2; Figure 2). The Muse S provided four-channel dry-electrode EEG, and the frontal channels AF7 and AF8 supplied the spectral summaries used here, including frontal theta, frontal alpha, and their ratio. Spectral features were computed on data rows after excluding event-marker rows and rows where the headband-contact flag indicated the device was off-head, following the preprocessing in Xu et al. (2025). The Muse S recording logged the device band-power stream rather than a raw waveform, so an external stage such as artifact subspace reconstruction could not be applied post-hoc. As the available check, the within-person association of frontal theta with stress was essentially unchanged after adjusting for the device-reported blink and jaw-clench rates (standardized β = 0.36 before and after; Supplementary Table S7). We used the on-device contact and blink flags for quality control, excluded off-head rows, and treat the frontal band powers as the device-level estimates that comparable consumer-EEG studies have validated (Krigolson et al., 2017; Cannard et al., 2021; Milstein and Gordon, 2020); dry frontal electrodes in a moving cockpit may nonetheless retain some non-neural contribution. The Polar H10 recorded beat-to-beat cardiac intervals, from which mean heart rate and beat-interval variability were computed (Shaffer and Ginsberg, 2017). The Embrace Plus recorded wrist EDA at 4 Hz and skin temperature at 1 Hz, and tonic and phasic EDA components were separated with neurokit2 (Makowski et al., 2021; Benedek and Kaernbach, 2010; Greco et al., 2016). The Pupil Labs Neon provided binocular gaze at 200 Hz with on-device fixation, saccade, and blink detection (Kassner et al., 2014; Carter and Luke, 2020). The Sentry Plus logged GPS-derived flight dynamics (ground speed, bank, pitch, g-load) at 1 Hz. Trial-level features were the maneuver means or variabilities of these signals.

**Table 2:** Consumer-grade devices deployed simultaneously during real flight.

Device	Modality	Sampling	Placement	Trial-level features used
Muse S Gen 2	EEG (4-ch dry)	~256 Hz	Forehead / temporal	Frontal (AF7, AF8) theta, alpha, beta, theta/alpha ratio; contact quality; device blink and jaw-clench rates
Polar H10	Cardiac (beat intervals)	Beat-to-beat	Chest strap	Mean heart rate, heart-rate SD, RR SD, ectopic-beat rate, beat-level SQI
Embrace Plus	EDA + skin temperature	4 Hz / 1 Hz	Non-dominant wrist	EDA mean, range, coefficient of variation, SCR rate and amplitude, phasic SD, tonic mean; temperature mean and range
Pupil Labs Neon	Eye movement	200 Hz	Glasses	Fixation, saccade, and blink rates; gaze sampling rate
Sentry Plus	Flight dynamics (GPS)	1 Hz	Side window	Ground speed (mean, SD), absolute bank (mean, SD), pitch SD, g-load (mean, SD), stationary fraction

The last column gives the full trial-level feature panel entered in the predictive models.

### 2.4 Signal-quality assurance

Because construct inference is only meaningful when the underlying signals are trustworthy, we first quantified signal quality in detail. As no comparable multimodal dataset exists for real general-aviation flight, this characterization is itself a primary deliverable of the study and not only a precondition. For each device and trial, we computed availability and modality-specific indicators: EEG headband-contact and per-channel good-contact rates and band-power validity; cardiac outlier and ectopic-beat rates and the Orphanidou signal-quality index applied to beat intervals (Orphanidou et al., 2015); EDA sampling stability, out-of-range fraction, and flat-signal and negative-value counts (Kleckner et al., 2018); eye worn-rate and effective sampling rate; and temperature and flight-dynamics sampling integrity. Each indicator carried an explicit pass rule: EEG contact good at a Muse horseshoe indicator of 2 or below (1-good to 4-no-contact) with valid band power on on-head rows; cardiac beats within 300 to 2000 ms with the largest gap below 3000 ms and a successive-interval ratio at or below 2.2; EDA within 0.01 to 100 microsiemens with no sustained flat or negative segments at 4 Hz; eye data with a worn-rate near unity and near 200 Hz sampling; and temperature within 20–42 degrees Celsius. Supplementary Table S1 gives every threshold with its trial-level pass rate. Three convergent checks, following recommendations for field-sensor evaluation (van Lier et al., 2020; Böttcher et al., 2022; Boucsein et al., 2012), summarized validity at the signal level: device-level benchmark comparison against published laboratory values, internal consistency of canonical physiology-behavior relationships, and physiological plausibility against normative ranges. This structure is complementary to concurrent-criterion protocols such as the three-level framework of van Lier et al. (2020), which require a simultaneous research-grade reference that the cockpit does not permit.

### 2.5 Analysis

The analysis followed the tiered logic directly. *Known-groups sensitivity* was assessed with Kruskal–Wallis tests of whether each outcome varied across the five maneuvers, with effect sizes reported as ϵ^2^. *Convergent evidence* was assessed with repeated-measures correlations (*r*_rm_; Bakdash and Marusich, 2017) between sensor features and self-reported state, which remove between-pilot variance and isolate the within-person relationship. *Criterion-related association* was assessed with ordinary least-squares models that regressed each outcome on a standardized sensor feature while including participant and maneuver fixed effects, with the small-cluster inference described in Section 2.6. We used linear models for the one-to-four ordinal instructor ratings as a linear-probability approximation that keeps these fixed effects interpretable; a proportional-odds re-estimation of the key associations agrees in sign (Supplementary Table S6). We chose participant fixed effects over random effects (cf. Barr et al., 2013), and because the fixed effects absorb most of the variance we report each tabled feature's increment in total variance explained (Δ*R*^2^) relative to a participant-plus-maneuver baseline, so the standardized β is not mistaken for the model's full explanatory power. Finally, to test generalization rather than association, we used grouped 5-fold cross-validation with held-out pilots, comparing a maneuver-only baseline against a maneuver-plus-sensors model for classification (logistic regression) and regression (ridge regression). The sensor panel was every trial-level feature in Table 2, and the baseline used maneuver indicators alone. For classification, each continuous outcome was dichotomized at its overall sample median, applied identically to both models so it does not bias their comparison; the continuous outcomes were also analyzed directly by ridge regression (Table 3 and Supplementary Table S5). Grouping was by pilot, so no pilot appeared in both training and test folds, and standardization and imputation were fit on training folds only.

**Table 3:** Cross-pilot prediction alongside a within-subject calibration analysis, both for the maneuver-plus-sensors model.

	Classification AUC		Regression *R*^2^	
Outcome	Cross-pilot	Within-subject	Cross-pilot	Within-subject
Stress	0.66	0.68	−0.56	0.07
Workload	0.43	0.54	−1.11	−1.32
Situation awareness	0.37	0.52	−10.7	−0.64
Technical performance	0.59	0.64	−2.20	−1.52
Non-technical performance	0.53	0.66	−5.93	−0.15

The cross-pilot columns are the generalization estimate, from grouped folds in which no pilot appears in both training and test. The within-subject columns come from shuffled folds in which a pilot's own trials enter training; they are optimistically biased by construction and bound what per-pilot calibration might recover, rather than estimating generalization (Section 2.5). The maneuver-only baseline AUC ranged from 0.56 to 0.75.

The predictive specification is as follows. Maneuver identity entered as one-hot indicators. Numeric sensor features were median-imputed and standardized, both fit on the training fold only; sensor cells were missing in approximately 9% of trial-feature entries, chiefly where a device dropped out mid-maneuver. Classification used L2-penalized logistic regression with inverse-frequency class weights, though the median split left classes close to balanced (51% to 68% positive), and regression used ridge. The submitted analysis fixed the penalties a priori at *C* = 0.5 and α = 10 without tuning. Since a fixed penalty could in principle handicap the larger sensor model, we repeated the comparison with the penalty selected by nested grouped cross-validation: an inner 4-fold split over training pilots only, searching *C*∈{0.01, 0.05, 0.1, 0.5, 1, 5} and α∈{0.1, 1, 10, 100, 1000}, so that no test-fold pilot entered a tuning decision. Uncertainty on the model comparison comes from the paired per-fold difference, as a percentile bootstrap interval over the five outer folds (Supplementary Table S9). To separate a genuine absence of information from a limit on cross-pilot transfer, we also ran the identical models under shuffled 5-fold cross-validation, in which a pilot's remaining trials can enter the training set. That scheme is not a generalization estimate: The same pilot appears in training and test folds, so the model inherits that pilot's baseline. We use it strictly as a calibration analysis, an upper bound on what per-pilot calibration might recover. With 40 pilots, this test is informative about direction but limited in power.

### 2.6 Multiple comparisons, selection, and small-cluster inference

The screens were broad: 33 features were tested against 9 outcome families for the repeated-measures correlations, and a 12-feature shortlist (ranked by within-person association) fed the fixed-effect models. We controlled the false discovery rate within each outcome family using the Benjamini–Hochberg procedure (Benjamini and Hochberg, 1995), denoting adjusted values *p*_adj_, and we note that correction was applied within, not across, families. Because the 12-feature fixed-effect shortlist was pre-screened on the same data by within-person association, those criterion associations are conditioned on the screen, are exploratory rather than confirmatory, and have not been validated on an independent sample.

Two features of this design make that caveat consequential enough to test directly rather than disclaim, so we ran three checks. First, because the shortlist and the fixed-effect test reuse the same data, we refit every model using all 33 prespecified features with Benjamini–Hochberg correction across the full unscreened set of 297 models, and we split the pilots at random into a screening half and an estimation half 200 times, forming the shortlist on the first half and estimating on the second (Supplementary Table S8). Second, inference rests on 33 to 40 pilot clusters of about four observations each, each carrying its own dummy. In that regime the conventional CR0 cluster-robust variance is severely downward biased, because the participant fixed effect absorbs most of the within-cluster residual before the sandwich estimator sees it. We therefore recomputed every tabled association with a leave-one-pilot-out (CR3-type) jackknife variance and with a restricted wild cluster bootstrap using Rademacher weights and 9,999 replications, recording how far each coefficient moves when a single pilot is deleted (Supplementary Table S4). Where CR0 and the bootstrap disagree we report the bootstrap. One label also needed correcting: the quantity printed as partial *R*^2^ was the increment in overall *R*^2^, a share of total variance, and is reported here as Δ*R*^2^. The conventional partial *R*^2^ runs roughly twice as large, so the relabeling makes the reported quantity smaller rather than larger. All analyses are exploratory. They describe construct-relevant structure in an observational field dataset and are not confirmatory tests (Barr et al., 2013). We therefore favor effect sizes and intervals over thresholds, and we read associations involving signal-quality indices as quality covariates rather than as evidence about cognitive state.

## 3 Results

### 3.1 Known-groups sensitivity

**Figure 3:**
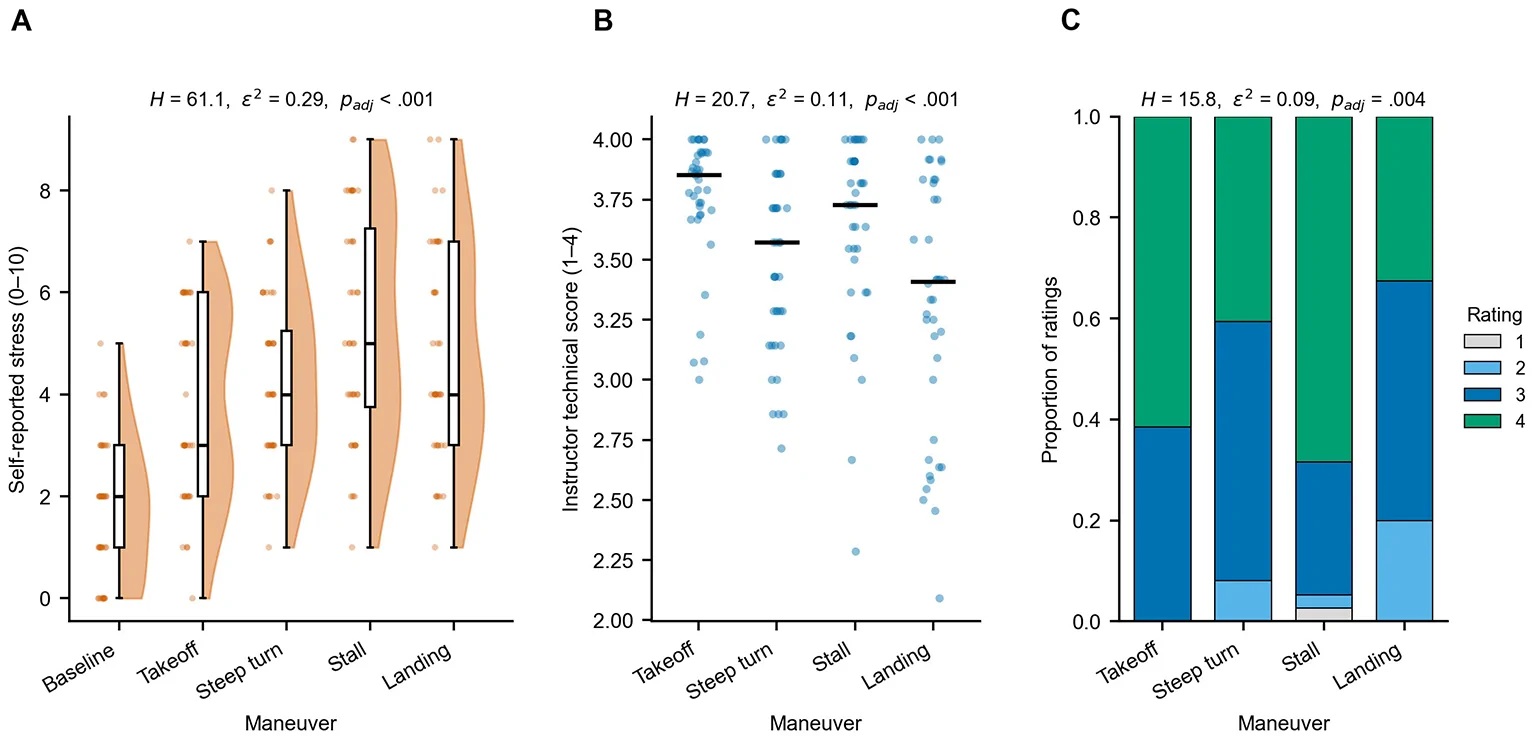
Known-groups sensitivity. Self-reported stress **(A)**, instructor-rated technical performance **(B)**, and instructor-rated workload management **(C)** vary systematically across maneuvers (Kruskal–Wallis *H*, ϵ^2^, and FDR-adjusted *p* annotated). Stress is shown as a distribution with individual trials overlaid; the ordinal instructor ratings are shown as proportional stacked bars so that ceiling effects remain visible.

The outcome labels carried structure imposed by the maneuvers (Figure 3). Self-reported stress rose from the ground Baseline through the more demanding maneuvers (Kruskal–Wallis *H* = 61.1, ϵ^2^ =.29, *p*_adj_ < .001, *n* = 200). Instructor-rated technical performance differed across maneuvers (*H* = 20.7, ϵ^2^ =.11, *p*_adj_ < .001, *n* = 160), as did instructor-rated workload management (*H* = 15.8, ϵ^2^ =.085, *p*_adj_ =.004, *n* = 154) and problem-solving (*H* = 10.7, *p*_adj_ =.031). Self-reported workload and situation awareness did not reach significance across this reduced maneuver set, so the known-groups precondition is met most clearly for stress and for the instructor scores. Workload and situation awareness likely call for protocols that vary these constructs more widely, a natural direction for future flights. What this tier can and cannot establish should be stated plainly. Because the maneuvers were flown in a largely fixed order, maneuver identity moves together with elapsed flight time, accumulating fatigue, the physical dynamics of the aircraft, and the settling of the sensors on the body. Any of these can produce graded variation in a physiological signal without cognitive demand playing a part. The known-groups result therefore shows that the outcome labels carry systematic structure, a precondition for everything that follows, but it cannot show that a signal is specific to stress or to workload.

### 3.2 Convergent evidence

**Figure 4:**
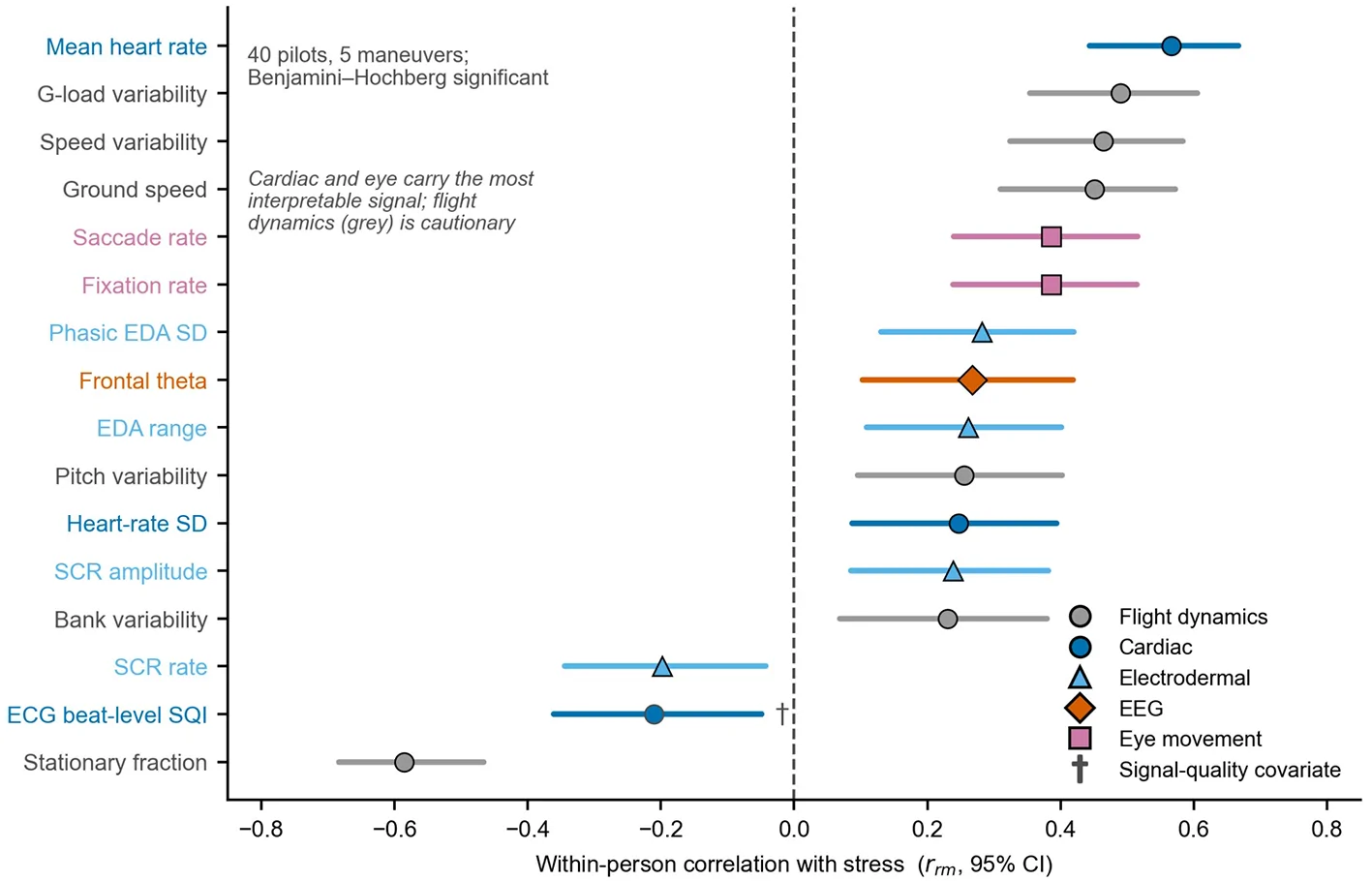
Within-person convergent validity. Repeated-measures correlations between sensor and flight-dynamics features and self-reported stress, restricted to features surviving false-discovery correction and colored by modality. Cardiac and oculomotor features carry the most interpretable physiological signal; the prominence of flight-dynamics features reflects that more demanding maneuvers are also more dynamic. Markers show *r*_rm_ with 95% confidence intervals.

Removing between-pilot differences revealed within-person convergence between sensor features and self-reported stress (Figure 4). Sixteen features survived false-discovery correction, and the most interpretable physiological signals were cardiac and oculomotor. Mean heart rate tracked stress most strongly among the physiological channels (*r*_rm_ =.57, 95% CI [.44, .67]), with beat-interval variability weaker (*r*_rm_ =.25). Saccade and fixation rates followed (*r*_rm_ =.39 each). Phasic EDA carried stress-related information that the tonic level did not: Phasic variability (*r*_rm_ =.28), EDA range (*r*_rm_ =.26), and skin-conductance response amplitude (*r*_rm_ =.24) were associated with stress, whereas trial-mean tonic EDA was not. Among EEG features, frontal theta band power tracked stress (*r*_rm_ =.27, *p*_adj_ =.006), and it was the only EEG feature to reach significance at this tier; the frontal theta/alpha ratio did not (all outcomes *p*_adj_>.53). Flight-dynamics features were among the strongest correlates overall (stationary fraction *r*_rm_ = −.59, g-load variability *r*_rm_ =.49, ground speed *r*_rm_ =.45). Their prominence is a caution as much as a finding: More demanding maneuvers are also more physically dynamic, so part of what the physiology tracks is the intensity of the maneuver rather than cognitive state alone. This convergent evidence is concentrated in the stress family. Workload and situation awareness, which varied less across this maneuver set, showed weaker within-person convergence here and are promising targets for protocols designed to elicit them.

### 3.3 Criterion-related association

**Figure 5:**
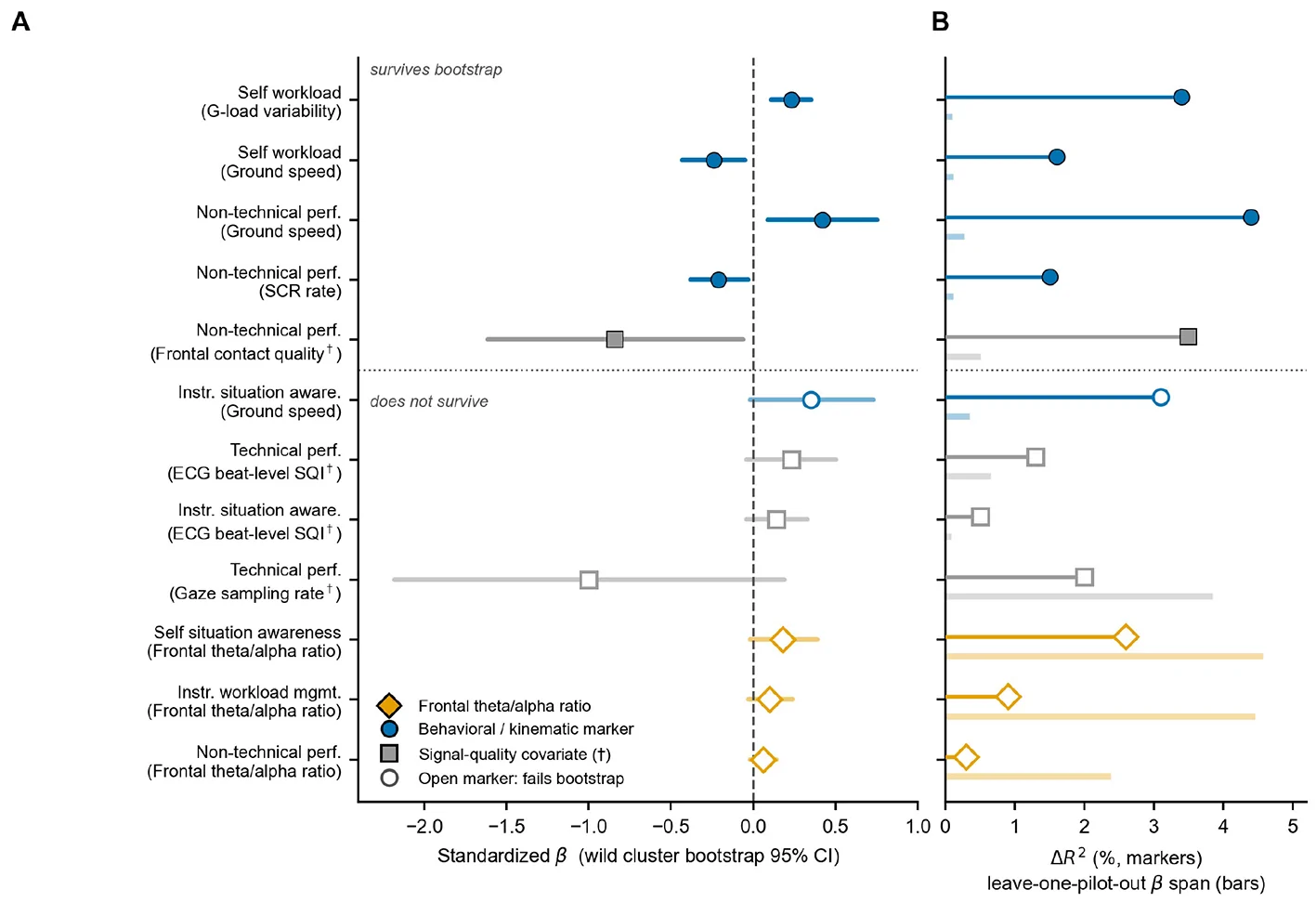
Criterion-tier associations for the main outcomes under robust small-cluster inference. **(A)** Standardized fixed-effect coefficients with wild cluster bootstrap 95% confidence intervals; filled markers survive the bootstrap, and open markers do not. **(B)** Increment in total variance beyond the fixed effects (Δ*R*^2^, markers), with the bar giving the leave-one-pilot-out coefficient range, rescaled for display. The frontal theta/alpha associations (diamonds) combine a small Δ*R*^2^ with an extreme leave-one-out range, the signature of an association carried by one pilot. Rows match Table 4.

The criterion tier adjusted for both pilot and maneuver and asked which features explain instructor-rated performance (Figure 5; Table 4). Three points govern the interpretation. First, the models carry high overall *R*^2^ (0.44 to 0.63), but this is absorbed almost entirely by the participant and maneuver fixed effects. Every tabled sensor feature added less than 5% of total variance beyond those effects (Δ*R*^2^, Table 4), so the standardized β describes a within-cell increment, not the model's explanatory power. Second, these models use 33 pilots and 4 maneuvers (Baseline has no in-air ratings), a smaller and different sample than the known-groups tier. Third, and decisively for what follows, the tier rests on 33–40 clusters of about four observations each. The conventional cluster-robust intervals reported in the original submission are therefore too narrow to be trusted, and Table 4 now reports wild cluster bootstrap intervals instead.

**Table 4:** Criterion tier: standardized associations between sensor features and rated outcomes, from models with participant and maneuver fixed effects (33–39 pilots, 4 maneuvers).

Outcome	Sensor feature	Std. β [bootstrap 95% CI]	*ΔR* ^2^	β range	*n*
Survives bootstrap inference					
Self workload	G-load variability	0.23 [0.11, 0.35]	.034	0.21, 0.26	145
Self workload	Ground speed	−0.24 [−0.43, −0.05]	.016	−0.25, −0.19	145
Non-technical performance	Ground speed	0.42 [0.09, 0.75]	.044	0.29, 0.43	144
Non-technical performance	SCR rate	−0.21 [−0.38, −0.03]	.015	−0.22, −0.16	154
Non-technical performance	Frontal contact quality †	−0.84 [−1.61, −0.06]	.035	−0.84, −0.58	132
Does not survive bootstrap inference					
Instructor situation awareness	Ground speed	0.35 [−0.02, 0.73]	.031	0.20, 0.38	144
Technical performance	ECG beat-level SQI †	0.23 [−0.04, 0.50]	.013	0.22, 0.56	143
Instructor situation awareness	ECG beat-level SQI †	0.14 [−0.04, 0.33]	.005	0.13, 0.17	142
Technical performance	Gaze sampling rate †	−1.00 [−2.18, 0.19]	.020	−1.01, +0.99	148
Self situation awareness	Frontal theta/alpha ratio	0.18 [−0.02, 0.39]	.026	0.18, 2.56	131
Instructor workload management	Frontal theta/alpha ratio	0.10 [−0.03, 0.24]	.009	0.09, 2.41	128
Non-technical performance	Frontal theta/alpha ratio	0.06 [−0.02, 0.14]	.003	0.05, 1.29	131

Intervals are wild cluster bootstrap intervals (Section 2.6). Δ*R*^2^ is the increment in total variance beyond the fixed effects, and the β range is the smallest to largest coefficient across leave-one-pilot-out refits. Selected main-outcome associations are shown; Supplementary Table S4 gives all 18 with the same diagnostics. Features marked † are signal-quality or device-artifact covariates, read as nuisance rather than cognitive-state evidence.

Under that corrected inference, the tier separates into two groups. Kinematic and electrodermal associations hold. G-load variability relates to self-reported workload (β =.23, bootstrap CI [.11, .35], Δ*R*^2^ =.034), ground speed to self-reported workload (β = −.24, [−.43, −.05]) and to non-technical performance (β =.42, [.09, .75]), and skin-conductance response rate negatively to non-technical performance (β = −.21, [−.38, −.03]). All four reproduce under both selection checks: refitting without the shortlist, and screening and estimating on disjoint halves of the pilots (Supplementary Table S8).

The frontal theta/alpha associations do not hold. In the original submission, the ratio was the one EEG feature to recur across outcomes with a consistent positive sign, relating to self-reported situation awareness (β =.18), instructor-rated workload management (β =.10), and non-technical performance (β =.06), each with a very narrow CR0 interval. The ratio is heavy-tailed here (skewness 8.8, largest value 156 times the median), and deleting the single pilot who contributes its extreme values moves the situation-awareness coefficient from 0.18 to 2.56. None of the three associations survives the bootstrap (*p* =.30, .38, and .41), and neither the unscreened refit nor the sample-splitting check recovers them. Since the ratio also failed to reach the convergent tier, this dataset provides no dependable criterion-tier evidence for it, and we have withdrawn the claim.

Several signal-quality indices also reached significance with large coefficients (frontal contact quality β = −.84 for non-technical performance; gaze sampling rate β = −1.00 for technical performance). These are not cognitive-state evidence. That they out-predict every cognitive feature points to a shared upstream confound, in which more demanding or longer maneuvers both degrade contact and sampling quality and attract lower scores. We flag them in Table 4 and interpret them as quality covariates. Robust inference divides them as it divides the cognitive features. Frontal contact quality survives for both non-technical performance (*p* =.030) and instructor-rated situation awareness (*p* =.013); gaze sampling rate reverses sign when one pilot is removed and does not survive (*p* =.21), nor does the beat-level cardiac index. That the one surviving quality covariate indexes electrode contact reinforces the shared-upstream-cause reading.

### 3.4 Cross-pilot generalization

**Figure 6:**
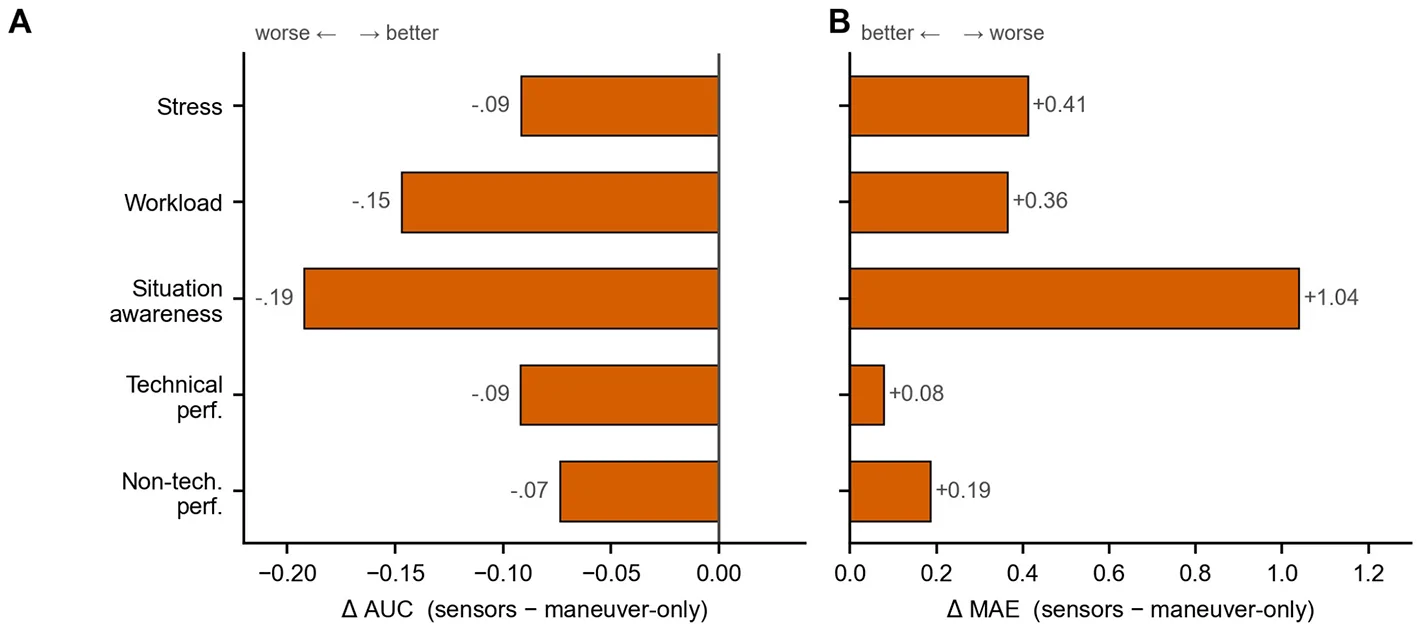
Explanatory-but-not-predictive boundary. Cross-pilot grouped cross-validation shown as the change from a maneuver-only baseline when sensor features are added. **(A)** Change in classification area under the curve (ΔAUC). **(B)** Change in regression mean absolute error (ΔMAE). Across all five outcomes ΔAUC is negative and ΔMAE is positive, so adding sensors did not improve, and often degraded, held-out performance, indicating that within-person associations do not yet transfer across pilots without calibration.

Within-sample association did not translate into cross-pilot prediction (Figure 6; Table 3). In grouped cross-validation with held-out pilots, adding the sensor panel did not exceed a maneuver-only baseline on these 40 pilots. For stress classification, area under the curve fell from .75 (maneuver only) to .66 (maneuver plus sensors); for workload from .57 to .43; for situation awareness from .56 to .37; for technical performance from .68 to .59; and for non-technical performance from .61 to .53. The regression models behaved the same way, with sensors increasing mean absolute error and driving cross-validated *R*^2^ below zero. Tuning the penalties by nested cross-validation inside each training fold does not change this. The direction is negative for every outcome. The fold-level bootstrap interval on the difference excludes zero for stress (ΔAUC −.08, 95% CI [−.11, −.05]), situation awareness (−.20, [−.37, −.03]), and technical performance (−.13, [−.17, −.09]); for workload and non-technical performance, it remains negative but spans zero (Supplementary Table S9). The penalty selected by the inner loop varied widely across folds, itself a sign that no stable predictive structure was available to tune toward.

The within-subject scheme supplies a different piece of information. Allowing a pilot's other trials into the training set, the same sensor panel recovered for stress and for the performance outcomes: Stress classification returned to .68 and non-technical performance to .66, and cross-validated regression *R*^2^ for stress rose from −.56 to .07. Workload and situation awareness stayed weak under both schemes. This is not a generalization estimate, and we do not present it as one. Trials from the same pilot appear in both the training and the test folds, so the model has access to that pilot's own baseline; the figures are optimistically biased and serve only as a calibration analysis, an upper bound on what per-pilot calibration might recover. The cross-pilot null is the deployment-relevant result. Read together, the two schemes are consistent with state-relevant information that is real but entangled with stable individual differences: usable once a model has seen the pilot, and not yet transferable to a new pilot without calibration. This explanation-to-prediction gap identifies individual calibration, personalized baselines, and larger pilot samples as the concrete route to pilot-general models. A similar combination of clear within-condition structure with as-yet limited cross-pilot transfer appears in Dehais et al. (2019).

### 3.5 Signal quality

The construct evidence rests on adequate signal quality (Table 5). Across the 200 nominal observations, trial-level availability ranged from 85% (EEG) to 97% (EDA and temperature), and complete data across all nine modality streams were present in 67% of trials. The one large internal quality gradient was EEG electrode location: Frontal channels held good contact (AF7 96%, AF8 94%), while temporal channels were much worse (TP9 73%, TP10 62%; Wilcoxon *W* = 805.5, *p* < 0.001, rank-biserial *r* = 0.57), which is why the analyses use frontal channels. Cardiac quality was high (beat-level signal-quality index 0.996; trial-level pass rate 81%; outlier rate 0.20%). EDA sampling was stable at 4.01 Hz with no flat or negative segments, eye tracking showed 99.9% worn-rate at 195 Hz effective sampling, and temperature was stable at 30.3°C. All eight physiological-plausibility checks passed at the sample-mean level. Signal quality showed no significant association with pilot age or flight experience, although with 40 pilots this null is low-powered (all |ρ| < 0.27, all *p*>0.10).

**Table 5:** Signal-quality foundation and three-step validation summary across the 200 nominal trial-participant observations.

Check	Key result	Status
Availability	85% (EEG) to 97% (EDA, temperature); 67% complete across nine streams	Adequate
EEG contact	Frontal 94–96%; temporal 62–73% (*W* = 805.5, *p* < .001, *r* =.57)	Frontal channels used
Cardiac quality	Beat-level SQI 0.996; trial-level pass 81%; outliers 0.20%	Benchmark-comparable
EDA/temperature	4.01 Hz, no flat or negative segments; temperature stable at 30.3°C	Benchmark-comparable
Eye movement	Worn-rate 99.9%; effective sampling 195 Hz	Benchmark-comparable
Internal consistency	5 of 8 canonical physiology-behavior pairs survive FDR (e.g., HR–stress *r*_rm_ =.57)	Supported
Physiological plausibility	8 of 8 indicators within normative ranges	Supported
Demographic moderation	No association of quality with age or flight hours (|ρ| < .27, low-powered)	None detected

## 4 Discussion

### 4.1 Main findings

Recorded inside an operating aircraft, multimodal wearable signals showed graded construct-relevant structure, but the strength of that evidence varied by construct and by modality. The clearest and most interpretable signal was the within-person coupling of cardiac and oculomotor activity with self-reported stress, supported by phasic EDA and by frontal theta band power. These associations are robust to false-discovery correction, biologically sensible, and consistent with decades of laboratory and simulator work (Roscoe, 1992; Hankins and Wilson, 1998; Wilson, 2002; Hughes et al., 2019; Peißl et al., 2018). The neural evidence is weaker than we judged it to be in the original submission. Frontal theta tracked stress at the convergent tier and survives control for the device-reported ocular and muscular proxies, which we retain. The frontal theta/alpha ratio does not survive: Its recurrence across instructor-rated outcomes turned out to depend on a single pilot and on a cluster-robust variance that is badly biased at these cluster sizes, and it failed the convergent tier to begin with. We therefore no longer present it as a candidate cockpit marker, and the EEG contribution of this dataset reduces to frontal theta at the convergent tier alone. The center of the paper is therefore 2-fold: Cardiac and oculomotor indices of stress carry the most usable within-person evidence in real flight, and the gap between explaining variance within pilots and predicting it across pilots maps out where the most valuable next work lies.

### 4.2 Theoretical interpretation

The findings sit within the neuroergonomics program of measuring the brain at work (Parasuraman, 2003) and its extension to integrated brain, body, and performance accounts of operational behavior (Mehta and Parasuraman, 2013). The theta/alpha rationale is grounded in the cognitive-neuroscience of its constituents: Frontal theta increases with cognitive control and working-memory load (Cavanagh and Frank, 2014; Klimesch, 1999), alpha desynchronizes with attentional engagement (Klimesch, 2012), and band ratios of this form have been related to self-reported workload (Raufi and Longo, 2022). That rationale did not survive contact with these data, and the reason is instructive for field work. A ratio of two band powers is unstable when the denominator approaches zero, which is exactly what dry frontal electrodes in a vibrating cockpit occasionally produce; the resulting heavy tail concentrated the association in one pilot. Ratio features of this kind need either a bounded transform or a prespecified outlier policy before they can bear weight in a field dataset, and we would recommend that to anyone building on this protocol. Two further limits temper the neural interpretation. We recorded lateral frontal channels (AF7 and AF8), not the midline site where frontal midline theta is classically generated, so our spectral summaries index lateral-frontal power and should not be equated with midline theta sources. The Muse S recording also logged device-computed band powers rather than raw samples, so no post-hoc ocular or EMG correction was possible; we mitigated this by verifying that the frontal theta association with stress is unchanged after adjusting for the device blink and jaw-clench rates (Supplementary Table S7), but a portion of the frontal spectral signal may still be non-neural.

### 4.3 Confounds

Two confounds shape the reading of the criterion tier. First, the strongest within-person correlates of stress are flight-dynamics features that index how physically dynamic a maneuver is, so physiology may partly track maneuver intensity rather than cognition. The participant and maneuver fixed effects reduce but do not remove this because within-maneuver kinematic variation still tracks demand. Second, signal-quality indices out-predict every cognitive feature in the criterion models. Rather than treat this as a finding, we read it as evidence of a shared upstream cause, in which harder or longer maneuvers degrade electrode and gaze contact and also attract lower instructor scores. This is exactly the kind of coupling that a construct-validity study must surface rather than bury. It bounds how far any single feature, neural or behavioral, can be read as a clean index of cognitive state. The bound differs by tier. Known-groups sensitivity is least affected because it asks only whether the labels vary across maneuvers. Convergent validity is affected where a within-person correlate is also quality-linked, which is why we keep the cardiac and oculomotor channels separate from the quality covariates. The criterion tier is most affected, since there the quality indices out-predict the cognitive features outright, so we read those associations as methodological covariates that constrain interpretation rather than as cognitive-state results.

### 4.4 Explanation vs. prediction

A particularly informative result is that adding sensors did not improve cross-pilot prediction on this sample, which we report directly rather than as a provisional shortfall. We read this as a marker of where the measurement currently sits and, more importantly, of how much room remains to develop it. The within-person correlations show that the signals carry state-relevant information. The cross-pilot result shows that this information is entangled with stable individual differences, which a pilot-general model can exploit once calibration is added. The same contrast appears in Dehais et al. (2019). Establishing how far a measure currently predicts is informative in its own right, and grouped cross-validation is a stringent test that field studies often omit. It also points clearly to the next step: Individual calibration, larger pilot samples, and personalized baselines are the concrete path toward field-deployable prediction. The contrast should not be over-read in the other direction either. The within-subject figures are optimistic by construction, so they establish that per-pilot information exists, not how much of it a calibrated system would actually capture.

### 4.5 Implications

For neuroergonomics, the study offers a template for field measurement. Verify signal quality as a precondition, build construct-relevant evidence in nested tiers, subject each tier to inference that suits the cluster structure actually available, weigh the result across modalities, and test generalization explicitly. The last two steps did real work here since they removed a finding we had been prepared to report. The explanatory-but-not-yet-predictive pattern is exactly the setting for passive braincomputer interfaces and operator-state monitoring (Zander and Kothe, 2011), which aim to track rather than command, and for which calibrated, individualized models are already the norm. Complementary field-portable neural imaging such as fNIRS (Gateau et al., 2015) may add spatial specificity beyond what lateral dry EEG provides. For aviation training specifically, the within-person sensitivity of low-cost cardiac and oculomotor measures means that physiological indicators may eventually complement instructor judgment during ordinary training, where ratings are observational and retrospective (Luzzani et al., 2024). A minimal cardiac-plus-eye configuration is the starting point the present evidence supports, and any such use would need reference measures stronger than the single-item self-reports and single-rater scores available here before it could be evaluated properly.

### 4.6 Limitations

Several constraints bound these conclusions. The data come from a single aircraft type under visual flight rules, so generalization to instrument flight, multi-crew, or turbine operations should be cautious. The maneuvers were flown in a largely standardized order, with only occasional weather-driven adjustments, so maneuver, elapsed time, and fatigue remain substantially confounded, and the known-groups result for stress is best read as task-or-time-graded. The direction of that bias is worth stating. The known-groups effect for stress may partly reflect time-on-task rather than maneuver demand, and the flight-dynamics correlates likely overstate the cognitive share of what the physiology tracks. None of the graded effects can be given a causal reading. The criterion models rest on 33 pilots and 4 maneuvers, a smaller sample than the headline design. Cluster-robust inference at this many clusters proved not merely borderline but actively misleading, which is why we report bootstrap intervals throughout and would caution field studies with similar cluster structure against the conventional estimator. The criterion tier that remains is small, and it is the tier we would most want an independent sample to confirm. Self-reports were single items and instructor ratings were 1–4 ordinal scales with ceiling effects, both of which attenuate and destabilize the small criterion effects. The instructor ratings also came from the single accompanying instructor on each flight, so inter-rater reliability could not be quantified in this dataset; the participant and maneuver fixed effects absorb stable rater tendencies but do not substitute for a formal reliability estimate, which future multi-rater or video-scored protocols should provide. Together, these reference measures are the binding constraint on how strong any claim here can be: Agreement with fallible indicators bounds rather than establishes the validity of a sensor feature. The 4 Hz wrist EDA limits phasic resolution (Boucsein, 2012; Greco et al., 2016). The sample was predominantly male, which bounds generalization, and wearable optical and physiological sensors in particular can show accuracy that varies across individuals and skin tones (Bent et al., 2020). Finally, all analyses are exploratory and observational; they identify construct-relevant structure, not causal pathways, and the cross-pilot results mark predictive deployment as a clear target for future work with individual calibration and larger samples.

## 5 Conclusion

In a real general-aviation cockpit, multimodal wearable signals showed graded within-person structure against task sequence and instructor-rated performance. The evidence is preliminary and construct-relevant rather than confirmatory: The reference measures are fallible indicators rather than criterion standards, the maneuver sequence confounds demand with elapsed time, fatigue, physical dynamics, and sensor settling, and the criterion tier rests on few pilot clusters. It is strongest for cardiac and oculomotor indices of stress, where within-person convergence is substantial and reproduces under sample splitting. Frontal theta adds weaker convergent support, while the frontal theta/alpha ratio did not withstand robust small-cluster inference and is not carried forward. Verified signal quality made these inferences admissible, and explicit cross-pilot testing, with tuning nested inside each training fold, showed that the sensor panel does not yet improve prediction in unseen pilots. The contribution we intend for field neuroergonomics is this first in-flight multimodal benchmark together with an explicit account of how far each modality's evidence currently reaches, including where it stops.

## Data Availability

The raw data supporting the conclusions of this article will be made available by the authors, without undue reservation.
