# Supplementary material for: From signal to construct evidence: multimodal consumer-wearable sensing in real general-aviation flight
Source: Front Neuroergon. 2026 Sep 1;7:1911259. doi: 10.3389/fnrgo.2026.1911259 (PMC13574616; doi:10.3389/fnrgo.2026.1911259)
Supplement: Supplementary file 1 [file Data_Sheet_1.pdf]

frontiers

Supplementary Material

1 SUPPLEMENTARY METHODS NOTE

All analyses use 40 pilots and five standardized maneuvers (Baseline, Normal Takeoff, Steep Turn, Power-on Stall, Normal Landing). The composite Circuit segment is excluded because its duration confounds maneuver effects with elapsed flight time. Within-person repeated-measures correlations (r_{rm}) follow Bakdash and Marusich (2017). Criterion models are ordinary least squares with participant and maneuver fixed effects. The Baseline segment carries no in-air ratings, so those models use four maneuvers. The false discovery rate was controlled with the Benjamini–Hochberg procedure within each outcome family. Rows marked $*$ rest on fewer than 20 pilots and should be read as exploratory. Features marked † are signal-quality or device-artifact covariates rather than cognitive-state indicators. Predictive models use grouped five-fold cross-validation with held-out pilots (logistic regression for classification, ridge regression for regression). Because the conventional CR0 cluster-robust standard error is downward biased with about four observations per pilot and a participant dummy per pilot, the criterion table (S4) reports leave-one-pilot-out and wild cluster bootstrap diagnostics alongside the CR0 intervals, and the bootstrap interval governs wherever the two disagree. Table S8 carries the two feature-selection sensitivity analyses, and Table S9 the cross-validation with hyperparameters tuned inside each training fold.

2 SUPPLEMENTARY TABLES

Table S1. Signal-quality metrics, thresholds, and results by modality. Thresholds are those applied in the analysis pipeline; results are the sample-level values reported in the main-text signal-quality table.

Modality	Metric	Threshold or pass rule	Result
EEG (Muse S)	Frontal contact (HSI)	HSI $\leq$ 2 on a 1-good to 4-no-contact scale	AF7 96%, AF8 94%
EEG (Muse S)	Band-power validity	finite estimate on on-head rows	85% availability
Cardiac (Polar H10)	Beat SQI (Orphanidou)	RR 300–2000 ms; max gap $\leq$ 3000 ms; RR ratio $\leq$ 2.2	SQI 0.996; trial pass 81%
Cardiac (Polar H10)	Outlier rate	RR outside 300–2000 ms	0.20%
EDA (Embrace Plus)	Range and continuity	0.01–100 microsiemens; no flat or negative runs; 4 Hz	no flat or negative; 4.01 Hz
Eye (Pupil Labs Neon)	Worn-rate and sampling	worn-rate near 1; effective rate near 200 Hz	99.9%; 195 Hz
Temperature (Embrace Plus)	Range	20–42 degrees Celsius; 1 Hz	stable 30.3 C

Table S2. Known-groups sensitivity: Kruskal–Wallis tests of whether each outcome varies across the five maneuvers, with Benjamini–Hochberg adjustment.

Outcome	H	ϵ^2	p_{adj}	n	Sig.
Self stress	61.12	0.293	<.001	200	yes
Self workload	5.45	0.016	.212	160	no
Self situation awareness	2.25	0.000	.588	160	no
Technical performance	20.67	0.113	<.001	160	yes
Non-technical performance	8.95	0.038	.054	159	no
Instructor situation awareness	2.71	0.000	.563	159	no
Instructor workload management	15.76	0.085	.004	154	yes
Problem solving	10.67	0.081	.031	99	yes
Leadership/teamwork	1.04	0.000	.791	47	no

Table S3. Within-person repeated-measures correlations between sensor or flight-dynamics features and self-reported state, for all associations surviving Benjamini–Hochberg correction. ∗ fewer than 20 pilots; † signal-quality covariate.

Outcome	Feature	r_{rm} [95% CI]	p_{adj}	n
Self stress	Stationary fraction	-0.58 [-0.68, -0.47]	<.001	178
Self stress	Mean heart rate	0.57 [0.44, 0.67]	<.001	179
Self stress	G-load variability	0.49 [0.35, 0.60]	<.001	178
Self stress	Speed variability	0.46 [0.32, 0.58]	<.001	178
Self stress	Ground speed	0.45 [0.31, 0.57]	<.001	178
Self stress	Saccade rate	0.39 [0.24, 0.52]	<.001	183
Self stress	Fixation rate	0.39 [0.24, 0.51]	<.001	183
Self stress	Phasic EDA SD	0.28 [0.13, 0.42]	.002	194
Self stress	EDA range	0.26 [0.11, 0.40]	.004	194
Self stress	Frontal theta	0.27 [0.10, 0.42]	.006	165
Self stress	Pitch variability	0.26 [0.10, 0.40]	.007	178
Self stress	SCR amplitude	0.24 [0.09, 0.38]	.007	194
Self stress	Heart-rate SD	0.25 [0.09, 0.39]	.007	179
Self stress	Bank variability	0.23 [0.07, 0.38]	.014	178
Self stress	ECG beat-level SQI†	-0.21 [-0.36, -0.05]	.025	179
Self stress	SCR rate	-0.20 [-0.34, -0.04]	.027	194
Technical performance	Temperature range	-0.38 [-0.52, -0.21]	<.001	155
Technical performance	Skin temperature	0.28 [0.10, 0.44]	.037	155

Table S4. Criterion-tier fixed-effect associations with robustness diagnostics, for every association that survived Benjamini–Hochberg correction on the conventional CR0 standard error. This is the complete list summarized in main-text Table 2. ∗ fewer than 20 pilots; † signal-quality or device-artifact covariate. The CR0 interval is the one reported in the original submission and is too narrow at these cluster sizes. The leave-one-pilot-out range is the smallest to largest coefficient obtained when any single pilot is deleted, and the bootstrap column is a restricted wild cluster bootstrap with Rademacher weights and 9,999 replications. An association whose leave-one-out range is wide relative to its coefficient is carried by individual pilots, which is what produced the narrow CR0 intervals; those associations do not survive the bootstrap. Rows are grouped by bootstrap survival and ordered within group by p.

Outcome	Feature	β [CR0 95% CI]	β leave-one-out range	Bootstrap 95% CI	p_{boot}	n
Survives the bootstrap						
Self workload	G-load variability	0.23 [0.10, 0.37]	0.21 to 0.26	0.11, 0.35	.001	145
Self workload	Device blink rate†	0.68 [0.19, 1.17]	0.01 to 0.64	0.16, 1.19	.011	136
Self workload	Ground speed	−0.24 [−0.43, −0.05]	−0.25 to −0.19	−0.43, −0.05	.012	145
Instructor situation awareness	Frontal contact quality†	−0.76 [−1.26, −0.25]	−0.80 to −0.48	−1.44, −0.07	.013	132
Non-technical performance	Ground speed	0.42 [0.20, 0.64]	0.29 to 0.43	0.09, 0.75	.014	144
Non-technical performance	SCR rate	−0.21 [−0.37, −0.05]	−0.22 to −0.16	−0.38, −0.03	.022	154
Non-technical performance	Frontal contact quality†	−0.84 [−1.18, −0.49]	−0.84 to −0.58	−1.61, −0.06	.030	132
Does not survive the bootstrap						
Instructor situation awareness	Ground speed	0.35 [0.09, 0.62]	0.20 to 0.38	−0.02, 0.73	.065	144
Technical performance	ECG beat-level SQI†	0.23 [0.10, 0.37]	0.22 to 0.56	−0.04, 0.50	.160	143
Technical performance	Gaze sampling rate†	−1.00 [−1.28, −0.72]	−1.01 to 0.99	−2.18, 0.19	.208	148
Leadership/teamwork ∗	ECG beat-level SQI†	1.11 [0.43, 1.79]	0.78 to 1.34	−0.36, 2.58	.247	46
Instructor situation awareness	ECG beat-level SQI†	0.14 [0.04, 0.24]	0.13 to 0.17	−0.04, 0.33	.290	142
Self situation awareness	Frontal theta/alpha ratio	0.18 [0.15, 0.21]	0.18 to 2.56	−0.02, 0.39	.303	131
Leadership/teamwork ∗	Frontal theta/alpha ratio	0.14 [0.08, 0.21]	−2.00 to 0.15	−0.07, 0.36	.357	45
Leadership/teamwork ∗	Gaze sampling rate†	4.66 [1.81, 7.51]	1.12 to 4.62	−2.57, 11.90	.379	42
Instructor workload management	Frontal theta/alpha ratio	0.10 [0.07, 0.14]	0.09 to 2.41	−0.03, 0.24	.382	128
Non-technical performance	Frontal theta/alpha ratio	0.06 [0.03, 0.09]	0.05 to 1.29	−0.02, 0.14	.413	131
Leadership/teamwork ∗	Frontal contact quality†	−1.08 [−1.92, −0.24]	−1.08 to 0.55	−3.75, 1.59	.471	46

3 SUPPLEMENTARY NOTE ON SMALL-SAMPLE AND SIGNAL-QUALITY RESULTS

Two outcomes rest on small pilot subsets: leadership/teamwork (13 pilots) and problem solving (36 pilots with sparse coverage). Associations involving them, including the frontal theta/alpha ratio for leadership/teamwork, are reported for completeness but should not be interpreted, because cluster-robust

Table S5. Cross-pilot prediction (grouped five-fold cross-validation with held-out pilots). Continuous outcomes were dichotomized at the overall sample median for classification. This table combines the classification and regression results summarized for the maneuver-plus-sensors model in main-text Table 3.

Classification (logistic regression)			
**Outcome**	**Model**	**AUC**	**Bal. acc.**
Self stress	Prevalence	0.50	0.50
Self stress	Maneuver only	0.75	0.70
Self stress	Maneuver + sensors	0.66	0.61
Self workload	Prevalence	0.50	0.50
Self workload	Maneuver only	0.57	0.57
Self workload	Maneuver + sensors	0.43	0.42
Self situation awareness	Prevalence	0.50	0.50
Self situation awareness	Maneuver only	0.56	0.53
Self situation awareness	Maneuver + sensors	0.37	0.45
Technical performance	Maneuver only	0.68	0.65
Technical performance	Maneuver + sensors	0.59	0.55
Non-technical performance	Maneuver only	0.61	0.61
Non-technical performance	Maneuver + sensors	0.53	0.55
Regression (ridge): mean absolute error and cross-validated R^2			
**Outcome**	**Model**	**MAE**	R^2
Self stress	Maneuver only	1.64	0.21
Self stress	Maneuver + sensors	2.05	-0.56
Self workload	Maneuver only	1.72	-0.20
Self workload	Maneuver + sensors	2.09	-1.11
Self situation awareness	Maneuver only	1.40	-0.03
Self situation awareness	Maneuver + sensors	2.44	-10.67
Technical performance	Maneuver only	0.32	0.11
Technical performance	Maneuver + sensors	0.40	-2.20
Non-technical performance	Maneuver only	0.56	-0.11
Non-technical performance	Maneuver + sensors	0.74	-5.93

Table S6. Proportional-odds ordinal-logistic robustness check for the ordinal instructor ratings. To keep the ordinal model estimable, both columns use a matched specification with maneuver fixed effects and a standardized feature, so participant fixed effects are omitted and the OLS betas differ from the main-text criterion models; the check targets the sign and significance of the feature. Signs agree in every row.

**Outcome**	**Feature**	**OLS β**	**OLS p**	**Ordinal coef.**	**Ordinal p**
Instructor workload management	Frontal theta/alpha ratio	0.041	$<.001$	1.375	.406
Instructor situation awareness	Ground speed	0.361	$<.001$	0.862	.004
Instructor situation awareness	Frontal theta/alpha ratio	0.066	$<.001$	0.342	.512
Problem solving / decision making	Frontal theta/alpha ratio	0.049	.005	0.609	.689
Instructor workload management	G-load variability	−0.114	.056	−0.326	.090

Table S7. EEG artifact-robustness check. The Muse S recording logged device band powers, not raw samples, so a raw-versus-preprocessed comparison could not be constructed; instead we test whether the within-person association of each frontal feature with self-reported stress survives control for the device-reported blink and jaw-clench rates, the on-device proxies for ocular and muscular activity. Models are OLS with participant fixed effects and cluster-robust standard errors; the feature and both covariates are standardized. Frontal theta is unchanged by the adjustment, indicating the association is not blink- or EMG-driven; the theta/alpha ratio is near zero regardless, consistent with its not reaching the convergent tier.

**Frontal feature**	**β unadjusted**	p	**β adj. blink + jaw**	p
Frontal theta	0.36	.006	0.36	.006
Frontal theta/alpha ratio	−0.01	.64	−0.00	.95

inference is unreliable below roughly 30 to 40 clusters. Signal-quality covariates (†) reach significance with large coefficients in the criterion tier (for example gaze sampling rate and frontal contact quality predicting performance scores). We interpret these as a shared upstream confound rather than cognitive-state evidence, in which more demanding or longer maneuvers degrade contact and sampling quality and also attract lower instructor scores.

Table S8. Feature-selection sensitivity. Panel A refits the criterion models using all 33 prespecified features rather than the 12-feature shortlist, with Benjamini–Hochberg correction applied within each outcome family across the full unscreened set of 297 feature-outcome models; the last two columns give the wild cluster bootstrap p and its BH adjustment for the eleven associations that survive on CR0. Panel B splits the pilots at random into a screening half and an estimation half 200 times, forms the shortlist on the screening half by within-person association only, and estimates the coefficient on the held-out half; selection rate is the proportion of splits in which the feature entered the shortlist, the interval is the 2.5th to 97.5th percentile of the held-out coefficient, and sign consistency is the proportion of splits sharing the modal sign. The two panels agree: kinematic and electrodermal associations reproduce under both checks, and every frontal theta/alpha association falls away under both.

A. All 33 prespecified features, no shortlist					
**Outcome**	**Feature**	β	p_{adj} **(CR0)**	p **(boot.)**	p_{adj} **(boot.)**
Self stress	RR interval SD	0.28	.009	.057	.057
Self stress	Heart-rate SD	0.18	.010	.042	.057
Self situation awareness	Frontal theta/alpha ratio	0.18	<.001	.300	.300
Instructor workload management	Frontal theta/alpha ratio	0.10	<.001	.394	.394
Leadership/teamwork∗	Frontal theta/alpha ratio	0.14	.045	.367	.367
Technical performance	Frontal theta/alpha ratio	0.08	.001	.408	.408
Technical performance	Gaze sampling rate†	−1.00	<.001	.213	.320
Technical performance	ECG beat-level SQI†	0.23	.020	.171	.320
Non-technical performance	Ground speed	0.42	.012	.012	.036
Non-technical performance	Frontal contact quality†	−0.84	.001	.026	.038
Non-technical performance	Frontal theta/alpha ratio	0.06	.017	.397	.397
B. Screening and estimation on disjoint halves of the pilots, 200 splits					
**Outcome**	**Feature**	**Sel. rate**	**Held-out** β	**2.5–97.5%**	**Sign consist.**
Self stress	Mean heart rate	1.00	0.19	−0.05, 0.45	.94
Self stress	Ground speed	1.00	−0.27	−0.71, −0.06	1.00
Self stress	G-load variability	1.00	0.11	−0.07, 0.29	.86
Self stress	Saccade rate	.97	0.11	−0.03, 0.26	.91
Self workload	G-load variability	.79	0.23	0.12, 0.34	1.00
Self workload	Ground speed	.58	−0.25	−0.48, −0.06	1.00
Self situation awareness	Frontal theta/alpha ratio	.65	0.15	−0.14, 0.38	.83
Instructor workload management	Frontal theta/alpha ratio	.80	0.14	−0.17, 0.45	.85
Non-technical performance	Frontal contact quality†	.84	−0.57	−1.26, −0.10	.99
Non-technical performance	Ground speed	.79	0.40	0.14, 0.65	.99
Non-technical performance	SCR rate	.52	−0.18	−0.31, −0.05	1.00
Non-technical performance	Frontal theta/alpha ratio	.60	0.10	−0.06, 0.38	.90

Table S9. Cross-pilot prediction with hyperparameters tuned by nested grouped cross-validation inside each training fold. The outer loop is grouped five-fold cross-validation with held-out pilots; the inner loop is grouped four-fold cross-validation over the training pilots only, selecting the logistic-regression penalty over $C \in \{0.01, 0.05, 0.1, 0.5, 1, 5\}$ and the ridge penalty over $\alpha \in \{0.1, 1, 10, 100, 1000\}$. Median imputation and standardization were fit on training folds only, and classes were balanced by inverse-frequency weighting. Intervals on the differences are percentile bootstrap intervals over the five outer folds. Nesting the tuning does not change the direction of any result: adding the sensor panel lowers held-out area under the curve and raises held-out error for every outcome. Sensor cells were missing in about 9% of trial-feature entries and the median split left classes near balance (51–68% positive).

**Outcome**	**AUC maneuver only**	**AUC + sensors**	**△AUC [95% CI]**	**△MAE [95% CI]**
Self stress	.75	.67	−.08 [−.11, −.05]	+0.21 [0.07, 0.38]
Self workload	.58	.42	−.16 [−.30, +.02]	+0.07 [0.01, 0.14]
Self situation awareness	.56	.36	−.20 [−.37, −.03]	+0.10 [0.04, 0.17]
Technical performance	.68	.55	−.13 [−.17, −.09]	+0.08 [−0.01, 0.16]
Non-technical performance	.61	.53	−.07 [−.26, +.11]	+0.02 [−0.01, 0.05]

Two features behave badly enough under leave-one-pilot-out deletion to warrant a specific note (Table S4). The frontal theta/alpha ratio is a ratio of two band powers and is severely heavy-tailed in this dataset (skewness 8.8, excess kurtosis 77.7, largest value 156 times the median). Deleting the single pilot who contributes the extreme values moves its coefficient for self-reported situation awareness from 0.18 to 2.56, and the corresponding coefficients for instructor workload management and non-technical performance rise by comparable factors. Gaze sampling rate reverses sign entirely, from -1.00 to $+0.99$, when one pilot is removed. In both cases the participant fixed effect absorbs almost all of the within-pilot residual for the influential pilot, which shrinks the CR0 meat matrix and produces the very narrow CR0 intervals. These associations are therefore not interpretable as sample-level effects, and we have removed them from

the claims made in the main text. The four leadership/teamwork rows behave the same way and fail the bootstrap outright, which confirms in inference what the cluster count already implied.
